# Responses to Comments of Weis

**DOI:** 10.1186/1477-7525-8-6

**Published:** 2010-01-15

**Authors:** Na Guo, Carlo A Marra, Fawziah Marra

**Affiliations:** 1 Collaboration for Outcomes Research and Evaluation (CORE), Faculty of Pharmaceutical Sciences, University of British Columbia, Vancouver, BC, Canada; 2 Centre for Health Evaluation and Outcome Sciences (CHEOS), Providence Health Care Research Institute, Vancouver, BC, Canada; 3 Faculty of Pharmaceutical Sciences, University of British Columbia, Vaccine and Pharmacy Services, British Columbia Centre for Disease Control (BCCDC), Vancouver, BC, Canada

## Abstract

A response to Weis and Pasipanodya 'Measuring health-related quality of life in tuberculosis: a systemic review - Response'.

## Letter to the Editor

We thank Weis and Pasipanodya for their valuable and insightful comments on our published manuscript, *Measuring health-related quality of life in tuberculosis: a systemic review *[1]. However, we want to address their question regarding our statement "A validated tuberculosis-specific quality of life instrument was not located". When we searched for a "validated tuberculosis-specific quality of life instrument" for our literature review, there were two requirements that we had determined apriori. Firstly, the quality of life (QoL) instrument had to be originally designed and tailored specifically to assess a tuberculosis (TB) population; and secondly, the involved QoL instrument had to be validated in various TB populations.

Subjective measurements, such as QoL, are always prone to inherent biases. Validation is an important process involving the accumulation of evidences to provide a sound scientific basis to support the inferences of their psychometric properties. In addition, the quality of validation evidences is very important. Although there is no rule of thumb when validating a QoL instrument, a number of considerations should be taken into account - for example reliability, validity and responsiveness, representative samples of the targeted population, appropriate analysis techniques, and controls over plausible confounding factors [2].

Through our literature review, we only found one QoL instrument, the DR-12 [3], which was designed specifically for use in TB infected populations. However, as we stated in our manuscript [1], it has only been used once in a sample of active TB patients and the validation was not done in a systematic fashion. More applications with better validation methods are needed to further establish the psychometric properties of the DR-12.

Finally, we agree with Weis and Pasipanodya that item banking is a promising direction for QoL measurements. Item banking is a large collection of items that are measuring the same health construct and calibrated onto common scales using item response theory (IRT) based approaches. A new generation of health outcomes measurements, computerized adaptive testing (CAT), which combines modern measurement theory, IRT, with advanced computer technologies, has the potential to optimize measurement precision [4-8].
